# In vitro screening for antiviral activity of Turkish plants revealing methanolic extract of *Rindera lanata* var. lanata active against human rotavirus

**DOI:** 10.1186/s12906-017-1560-3

**Published:** 2017-01-24

**Authors:** Andrea Civra, Rachele Francese, Davide Sinato, Manuela Donalisio, Valeria Cagno, Patrizia Rubiolo, Ramazan Ceylan, Ahmet Uysal, Gokhan Zengin, David Lembo

**Affiliations:** 1 Department of Clinical and Biological Sciences, University of Torino, S. Luigi Gonzaga Hospital, Regione Gonzole, 10, 10043 Orbassano, Torino Italy; 2 Dipartimento di Scienza e Tecnologia del Farmaco, Università degli Studi di Torino, 10125 Torino, Italy; 3 Department of Biology, Science Faculty, Selcuk University, Konya, Turkey; 4 Deparment of Medicinal Laboratory, Vocational School of Health Services, Selcuk University, Konya, Turkey

## Abstract

**Background:**

Human rotavirus (HRoV) is the leading cause of severe gastroenteritis in infants and children under the age of five years. No specific antiviral drug is available for HRoV infections and the treatment of viral diarrhea is mainly based on rehydration and zinc treatment. In this study, we explored medicinal plants endemic to Turkey flora as a source of anti-HRoV compunds.

**Methods:**

We performed an antiviral screening on *Ballota macrodonta*, *Salvia cryptantha* and *Rindera lanata* extracts by focus reduction assay. The extract with the highest selectivity index (SI) was selected; its antiviral activity was further confirmed against other HRoV strains and by virus yield reduction assay. The step of viral replicative cycle putatively inhibited was investigated by in vitro assays.

**Results:**

The methanolic extract of *R. lanata* (Boraginaceae) showed the most favourable selectivity index. This extract exhibited a dose-dependent inhibitory activity against three different HRoV strains (EC_50_ values ranging from 5.8 μg/ml to 25.5 μg/ml), but was inactive or barely active against other RNA viruses, namely human rhinovirus and respiratory syncytial virus. The *R. lanata* extract targets the early steps of HRoV infection, likely by hampering virus penetration into the cells.

**Conclusion:**

These results make the *R. lanata* methanolic extract a promising starting material for a bioguided-fractionation aimed at identifying anti-HRoV compounds. Further work is required to isolate the active principle and assess its clinical potential.

## Background

Gastroenteritis accounts for almost 20% of the deaths of children under five years (National Collaborating Centre for Women’s and Children’s Health. 2012). Viruses cause approximately 70% of acute gastroenteritis, with rotavirus infection frequently implicated in cases that require hospitalization [1, 2]. Human rotavirus (HRoV, family Reoviridae, genus Rotavirus) is responsible for 114 million episodes of diarrhea, 25 million clinic visits, 2.4 million hospital admissions, and more than 500,000 deaths in children up to age 5 occur worldwide annually [3]. While most of the episodes are mild, about 10% of cases lead to dehydration requiring a doctor visit, and in resource-constrained nations, one in 250 children will die from this dehydration [4–7]. In Europe, HRoV infection accounts for more than 50% of hospitalizations for gastroenteritis and about one-third of emergency department visits [1, 4, 8]. At present, the treatment of viral diarrhea is based on fluid replacement and zinc treatment in order to prevent dehydration and to decrease the severity and duration. So far, no specific antiviral drug is available to treat HRoV infections. Discovering novel, safe and effective antiviral molecules is a difficult task. One strategy is to explore plants as a source of novel active compounds due to the large structural diversity of natural products. Indeed, several studies reported on anti-HRoV activity by plant extracts [9–14]. In this context, we performed an antiviral screening of three plants endemic to Turkey flora and here we report on the anti-HRoV activity of *Rindera lanata* (Boraginaceae). Boraginaceae is a plant family represented by about 2,000 species in the world [15]. The genus *Rindera* includes about 25 species distributed mainly in Central and Western Asia [16], four of which growing in Turkey, namely *R. lanata*, *R. caespiota*, *R. albida* and *R. dumani. Rindera* species are widely known as “Yünlü gelin” and used as an anti-inflammatory agent in Anatolian folk medicine [17]. *R. lanata* is used to alleviate joint pains in Iranian folk medicine [18]. However, to the best of our knowledge, *R. lanata* was neither traditionally used nor scientifically investigated for antiviral properties. The present study reports the anti-HRoV potency, the spectrum of antiviral activity, and the probable mechanisms of antiviral action of the *R. lanata* methanol extract.

## Methods

### Chemicals

Quinic acid (>98%), malic acid (>99%), cholorogenic acid (>95%), protocatechuic acid (>97%), caffeic acid (>98%), *p-*coumaric acid (>98%), rosmarinic acid (>96%), rutin (>94%), hesperidin (>97%) and hyperoside (>97%) were purchased from Sigma-Aldrich (Germany). Apigenin (>99%) and rhamnetin (>99%) were purchased from Fluka (Germany).

### Plant material and extraction

Taxonomic identification of the plant materials were confirmed by the senior taxonomist Dr. Murad Aydin Sanda, from the Department of Biology, Selcuk University. The voucher specimens were deposited at the Konya Herbarium of Department of Biology, Selcuk University, Konya-Turkey. *Rindera lanata* (Lam.) Bunge var. *lanata* (Lam.) Bunge (family: Boraginaceae) was collected from Balcilar-Taskent, Konya-Turkey, May 2014, (Voucher No: GZ 1410). *Salvia cryptantha* Montbret et Aucher ex Bentham (family: Lamiaceae) was provided by Selcuk University, Aladdin Keykubat Campus, Yukselen village- Campus road, Konya-Turkey, June 2014, (Voucher No: GZ 1421). *Ballota macrodonta* Boiss. et Bal (family: Lamiaceae): was collected from Camardi, Mazmili Mountain, Nigde-Turkey, June 2014 (Voucher No: 1425).

The plant materials were dried at room temperature. The air-dried aerial parts (leaves, stem, flowers) were collected as a mix (10 g) and were macerated with 250 mL of methanol at room temperature for 24 h. Methanol was then removed with a rotary evaporator at 40 °C. The extracts were stored at +4 °C until analyzed. Yield of these extracts were 17.83%, 14.86% and 4.90%, respectively.

For both cell viability assays and infectivity assays, the extracts were reconstituted in sterile methanol, to a concentration of 25 mg/ml. Every experiment was performed with freshly resuspended extracts.

### LC-MS/MS analysis

LC-MS/MS analyses of the phenolic compounds were performed on a Shimadzu Nexera UHPLC system coupled to a triple quadrupole LC-MS8040 system equipped with an ESI source operating in both positive and negative ionization modes. The liquid chromatograph was equipped with LC-30 AD binary pumps, DGU-20A3R degasser, CTO-10ASvp column oven and SIL-30 AC autosampler. The chromatographic separation was performed on a C18 reversed-phase Inertsil ODS-4 (150 mm × 4.6 mm, 3 *μ*m particle size, GL Sciences, The Netherlands) analytical column. The column was maintained at 40 °C. The elution gradient consisted of mobile phase with eluent A (water, 5 mM ammonium formate and 0.1% formic acid) and eluent B (methanol, 5 mM ammonium formate and 0.1% formic acid). The mobile phase gradient was as follows; from 40% to 90% eluent B in 20 min, then 90% B for 4 min. The flow rate of the mobile phase was 0.5 mL/min and the injection volume 4 μL.

MS conditions were: interface temperature; 350 °C, DL temperature; 250 °C, heat block temperature; 400 °C, nebulizing gas flow (Nitrogen); 3 L/min and drying gas flow (Nitrogen); 15 L/min. LC-MS/MS data were collected and processed by LabSolutions software (Shimadzu, Kyoto, Japan). The multiple reaction monitoring (MRM) mode was used to quantify the analytes by adopting two or three transitions per compound, the first one for quantitative purposes and the second and/or the third one for confirmation. These analytical conditions have been successfully used to analyze other plant matrices [19–21].

### Cell lines and viruses

African green monkey kidney epithelial cells (MA104) and human epithelial adenocarcinoma HeLa cells (ATCC® CCL-2) were propagated in Dulbecco’s Modified Eagle Medium (DMEM; Sigma, St. Louis, MO) supplemented with 1% (v/v) Zell Shield (Minerva Biolabs, Berlin, Germany) and heat inactivated, 10% (v/v) fetal bovine serum (Sigma). Human epithelial cells HEp-2 (ATCC® CCL-23) were grown as monolayers in Eagle’s minimal essential medium (MEM) (Gibco/BRL, Gaithersburg, MD) supplemented with 10% FBS and 1% antibiotic-antimycotic solution. HRoV strains Wa (ATCC® VR-2018), HRoV 408 (ATCC® VR-2273) and DS-1 (ATCC® VR-2550) were purchased from ATCC; virus was activated with 5 μg/ml of porcine pancreatic trypsin type IX (Sigma, St. Louis, Mo.) for 30 min at 37 °C and propagated in MA104 cells by using DMEM containing 0.5 μg of trypsin per ml as described previously [22]. Respiratory Syncytial Virus (RSV) strain A2 (ATCC® VR-1540) was propagated in HEp-2 and titrated by the indirect immunoperoxidase staining procedure using an RSV monoclonal antibody (Ab35958; Abcam, Cambridge, United Kingdom) as described previously [23]. Human rhinovirus (HRhV) 1A (ATCC® VR-1559) was cultured in HeLa cells, at 34 °C, in a humidified 5% CO_2_ incubator. When the full cytopathic effect (CPE) developed, cells and supernatants were harvested, pooled, frozen and thawed three times, clarified and stored at −70 °C. HRhV titers were determined by the standard plaque method as described previously [22].

### Cell viability assay

Cells were seeded at a density of 5 × 10^3^/well in 96-well plates and treated the following day with serially-diluted methanol extracts of *B. macrodonta*, *S. cryptantha* or *R. lanata* (1.2 μg/ml to 1666 μg/ml) to generate dose–response curves. Control wells (100% of viability) were prepared by treating cells with equal volumes of methanol, corresponding to 6.7% (v/v) to 0.0048% (v/v) in cell media. After 24 h of incubation, cell viability was determined using the CellTiter 96 Proliferation Assay Kit (Promega, Madison, WI, USA), and following the manufacturer’s instructions. Absorbances of both treated (Abs_T_) and untreated (Abs_METH_) samples were measured using a Microplate Reader (Model 680, BIORAD) at 490 nm. The % of cell viability was calculated according to the following formula: (Abs_T_ X 100)/Abs_METH_. The 50% cytotoxic concentration (CC_50_) was determined using logarithmic viability curves. Where possible, a selectivity index (SI) was calculated by dividing the CC_50_ by the EC_50_ value.

### Rotavirus inhibition assays

The anti-HRoV efficacy of methanol extracts of *B. macrodonta*, *S. cryptantha* and *R. lanata* was determined by focus reduction assay. Assays of inhibition of rotavirus infectivity were carried out with confluent MA104 cell monolayers plated in 96-well trays, as described elsewhere [22]. Cells were treated for 2 h at 37 °C with methanol extract of *R. lanata*, at concentrations ranging from 1.2 to 300 μg/ml in serum-free medium prior to virus addition. HRoV infection was performed at a multiplicity of infection (MOI) of 0.02 for 1 h at 37 °C, in presence of the methanol extract unless otherwise stated. Infected cells were washed with serum-free medium, fresh methanol extract was added, and cells were incubated in this medium at 37 °C in a humidified incubator in 5% (vol/vol) CO_2_–95% (vol/vol) air. For time of addition assays, serial dilutions of extracts were added on cells alternatively 2 h before infection or during infection or post infection. Control samples were prepared by treating cells with culture medium supplemented with equal volumes of methanol and taken as 100% of infection. After 16 h of incubation, infected cells were fixed with cold acetone-methanol (50:50), and viral titers determined by indirect immunostaining by using a mouse monoclonal antibody directed to human rotavirus VP6 (0036; Villeurbanne, France), and the secondary antibody peroxidase-conjugated AffiniPure F(ab’)_2_ Fragment Goat Anti-Mouse IgG (H + L) (Jackson ImmunoResearch Laboratories Inc., 872 W. Baltimore Pike, West Grove, PA 19390). Blockade of viral infectivity is expressed as mean % ± SD. Where possible, anti-viral effective concentration (EC_50_) values were calculated by regression analysis using the dose–response curves generated from the experimental data, using PRISM 4 (GraphPad Software, San Diego, CA, U.S.A.).

### RSV inhibition assay

HEp-2 cells were first seeded (at 8 × 10^3^ cells/well) in 96 well plate. The methanol extract of *R. lanata* was serially diluted in medium (from 300 to 0.4 μg/ml) and incubated with cells for 2 h at 37 °C, then mixtures of extracts and virus (MOI 0.01) were added to cells to allow the viral adsorption for 3 h at 37 °C; the monolayers were then washed and overlaid with 1.2% methylcellulose medium containing serial dilutions of extract. Control wells (100% of infection) were prepared by treating cells with equal volumes of methanol. Three days post-infection, cells were fixed with cold methanol and acetone (50:50) for 1 min and subjected to RSV-specific immunostaining. Immunostained plaques were counted, and the percent inhibition of virus infectivity was determined by comparing the number of plaques in treated wells with the number in untreated control wells.

### Rhinovirus inhibition assay

HeLa cells were first seeded (at 8 × 10^4^ cells/well) in 24 well plates. 24 h later the methanol extract of *R. lanata* was serially diluted in medium (from 300 μg/ml to 0.4 μg/ml) and added to cell monolayers. After 2 h of incubation (37 °C, 5% CO_2_), medium was removed and infection was performed with 200 μL/well with HRhV 1A (MOI 0.0002) and different concentrations of the extract. The infected cells were incubated at 34 °C for 1 h, then washed with medium, and overlaid with a 1:1 combination of 1.6% SeaPlaque Agarose and 2X DMEM containing the extract. Control wells (100% of infection) were prepared by treating cells with equal volumes of methanol. The plates were incubated at 34 °C for 3 days. After incubation, the plates were fixed with 7.5% formaldehyde (Fluka) and stained with crystal violet (Sigma, St. Louis, Mo.). The number of plaques formed was counted.

### Assay of rotavirus yield

To test the ability of *R. lanata* extract to inhibit multiple cycles of HRoV replication, confluent MA104 cells in 24-well trays were infected with trypsin-activated Wa rotavirus (MOI 0.02) for 1 h at 37 °C and washed as above. Cells were incubated in medium supplemented with 0.5 μg/ml porcine trypsin and 1.2, 3.6, 11, 33, 100, 300 μg/ml of *R. lanata* extract. Infected cells and cell supernatants were harvested at 48 h post infection and virus titers determined by indirect immunostaining of MA104 cell monolayers inoculated with serial dilutions of the samples. The assay was performed in triplicate. One-way analysis of variance (ANOVA), followed by the Bonferroni test, was used to assess the statistical significance of differences in virus titers. Significance was set at 95% level.

### Virus inactivation assay

Trypsin-activated HRoV Wa was incubated for 2 h at 4 °C in presence of 100 μg/ml of *R. lanata* extract. After this incubation, both treated and untreated viruses were titrated. Viral yields were determined by indirect immunostaning, by counting the number of infective events at dilutions at which the extract was no more active (i.e. 1:1024, 1:2048, 1:4096).

### Entry assays

In order to test the effect of *R. lanata* methanol extract on the rotavirus-cell penetration process, an entry assay was performed. Briefly, MA104 cells were cultured to confluence in 96-well trays; cells were washed twice with DMEM and then cooled on ice for 20 min. Activated virus, which had been cooled to 4 °C, was allowed to attach to cells on ice for 1 h at 4 °C, at a MOI of 0.02. Cells were washed three times, then *R. lanata* methanol extract was added to the cells (at concentrations ranging from 1.2 μg/ml to 300 μg/ml) and virus particles were allowed to penetrate inside cells for 1 h at 37 °C. The entry of the viral particles was stopped with two quick washes with 3 mM EGTA in PBS, which releases the outer layer of the virions and causes the particles to detach from the cell surface [24, 25]; finally, cells were incubated with warm DMEM. After 16 h, cells were fixed and blockade of virus entry were determined by focus reduction assay as described above, and expressed as mean percentages of untreated control ± SEM. Significant differences were assessed by one way ANOVA, using PRISM 4 (GraphPad Software, San Diego, California, USA).

### Rotavirus cell-binding assay

Confluent MA104 cell monolayers in 24-well trays were washed, incubated with *R. lanata* methanol extract for 2 h at 37 °C and cooled on ice for 20 min in the continuing presence of the extract. Trypsin-activated virus, which had been cooled to 4 °C, was allowed to attach to cells on ice for 1 h (MOI = 3) in presence of *R. lanata* methanol extract. Cells were washed with cold DMEM, followed by addition of cold DMEM. Cells were subjected to two rounds of freeze-thawing, incubated at 37 °C for 30 min with 10 μg/ml porcine trypsin to release bound virus, and the lysate clarified by low speed centrifugation for 10 min. Cell-bound virus titers were determined by indirect immunostaining as above. Significant differences were assessed by Student’s *t*-test, using PRISM 4 (GraphPad Software, San Diego, California, USA).

## Results

### *R. lanata* methanolic extract is endowed with anti-HRoV activity

In a first set of experiments, we tested the anti-HRoV activity of *R. lanata, B. macrodonta* and *S. cryptantha* methanolic extracts against HRoV Wa. Results depicted in Table 1 show that all the methanolic extracts were endowed with anti-HRoV activity, with EC_50_s ranging from 5.8 to 16.3 μg/ml. To exclude the possibility that the antiviral activity might depend on a cytotoxic effect, cell viability assays were performed on uninfected cells, challenged with the extracts under the same conditions as the antiviral assays. Control wells were prepared by treating cells with equal volumes of methanol, corresponding to 6.7% (v/v) to 0.0048% (v/v) in cell media: these low volumes of methanol were non cytotoxic and did not influence viral replication (data not shown). As shown in Table 1, *R. lanata* showed the most favourable selectivity index (SI), i.e. 205. Therefore, we focused our research on *R. lanata* methanolic extract. Firstly, we performed LC-MS/MS analysis to identify and quantify phenolic components that characterize *R. lanata* methanolic extract, in order to make sure of the reproducibility of our study and to ensure that the results of future studies will be comparable to the ones of this research. The LC-MS/MS analysis allowed to identify and quantify twelve phenolic compounds in *R. lanata* methanolic extract and the results are listed in Table 2. Malic acid (9381.66 μg/g extract) and quinic acid (2318.60 μg/g) were found to be major phenolic component followed by rosmarinic acid (706.38 μg/g) hesperidin (399.74 μg/g extract), *p-*coumaric acid (173.59 μg/g extract), and hyperoside (118.00 μg/g extract). In accordance with these results, *R. lanata* belongs to the Boraginaceae family, characterized by the presence of rosmarinic acid and related components [26]

**Table 1:** Antiviral activity of *R. lanata*, *B. macrodonta* and *S. cryptantha* methanolic extract against HRoV Wa

HRoV strain	Source	EC50^a^ (μg/ml) – 95% C.I.^b^	CC50^c^(μg/ml)	SI^d^
Wa	*Salvia cryptantha*	16.3 (9.0–29.6)	134.8	8.3
	*Ballota macrodonta*	8.1 (6.9–9.7)	445.5	55
	*Rindera lanata*	5.8 (4.6–7.2)	1179	205

^a^EC_50_ half maximal effective concentration

^b^C.I. confidence interval

^c^CC_50_ half maximal cytotoxic concentration

^d^SI selectivity index

**Table 2:** Quantification of identified phenolic compounds in methanol extract of *Rindera lanata* by LC ESI MS/MS in negative ionization mode

Analytes	RT	[M-H]^−^(m/z)	MS^2^ (m/z)	Equation	Amount^a^
Quinic acid	3.36	190.95	85, 93	*f(x) =* 33.66x + 25133	2318.60 ± 111.29
Malic acid	3.60	133.05	115, 71	*f(x) =* 93.61x-5673	9381.66 ± 497.23
Chlorogenic acid	5.29	353	191	*f(x) =* 48.98x + 26780	23.63 ± 1.16
Protocatechuic acid	5.51	152.95	109, 108	*f(x) =* 36.86x + 6197	32.3 ± 1.65
Caffeic acid	7.11	178.95	135, 134, 89	*f(x) =* 1585x + 83958	13.53 ± 0.70
*p*-Coumaric acid	9.17	162.95	119, 93	*f (x) =* 73.53x + 27064	173.59 ± 8.85
Rosmarinic acid	9.19	358.9	161, 133	*f (x) =* 18.02x + 1149	706.38 ± 34.61
Rutin	9.67	609.1	300, 271, 301	*f(x) =* 51.88 + 3841	101.89 ± 5.09
Hesperidin	9.69	611.1	303, 465	*f(x) =* 1975.77 + 105641	399.74 ± 19.59
Hyperoside	9.96	463.1	300, 301	*f(x) =* 0.98x + 827	118.00 ± 5.78
Apigenin	16.73	268.95	151, 117	*f(x) =* 543.79 + 18525	0.10 ± 0.005
Rhamnetin	18.41	314.95	165, 121, 300	*f(x) =* 110.09 + 632.44	0.23 ± 0.014

RT: Retention time

[M-H]^−^ *:* Deprotonated ions of the standard compounds

MS^2^: MRM fragments for the related molecular ions

^a^Values in *μ*g/g (w/w) of plant methanol extract

To assess the spectrum of anti-HRoV activity, the extract was tested against two additional HRoV strains, namely HRoV 408 and DS-1. The results reported in Table 3 clearly show that the extract was effective in inhibiting the infectivity of both strains tested, although to a different extent, with EC_50_ values of 25.5 μg/ml and 14.3 μg/ml respectively. The specificity of the anti-HRoV activity was assessed by performing antiviral assays against two unrelated viruses, namely the rhinovirus (HRhV) and the respiratory syncytial virus (RSV). These were selected for being both RNA viruses as HRoV and representative of naked (HRhV) or enveloped (RSV) viruses. The methanolic extract of *R. lanata* did not show any antiviral activity against HRhV (Table 4) while it was possible to calculate an EC_50_ against RSV (45.3 μg/ml). However, the selectivity index was very low (4), suggesting that the anti-RSV activity was mainly an expression of a cytotoxic effect (Table 4). Taken together, the results presented so far demonstrate that the *R. lanata* methanolic extract exerts a specific anti-HRoV effect which is not strain restricted.

**Table 3:** Antiviral activity of *R. lanata* methanolic extract against HRoV strains

HRoV strain	EC_50_^a^ (μg/ml) – 95% C.I.^b^	CC_50_^c^(μg/ml)	SI^d^
HRoV408	25.5 (15.6–41.6)	1179	46
DS-1	14.3 (6.7–30.2)	1179	83

^a^EC_50_ half maximal effective concentration

^b^C.I. confidence interval

^c^CC_50_ half maximal cytotoxic concentration

^d^SI selectivity index

**Table 4:** Antiviral activity of *R. lanata* methanolic extract against HRhV 1A and RSV A2

Virus	EC_50_^a^ (μg/ml) – 95% C.I.^b^	CC_50_^c^(μg/ml)	SI^d^
HRhV 1A	n.a.	804.3	n.a.
RSV A2	45.28 (30.20–67.88)	183.9	4.1

^a^EC50 half maximal effective concentration

^b^C.I. confidence interval

^c^CC50 half maximal cytotoxic concentration

^d^SI selectivity index

n.a. not assessable

**Fig. 1:**
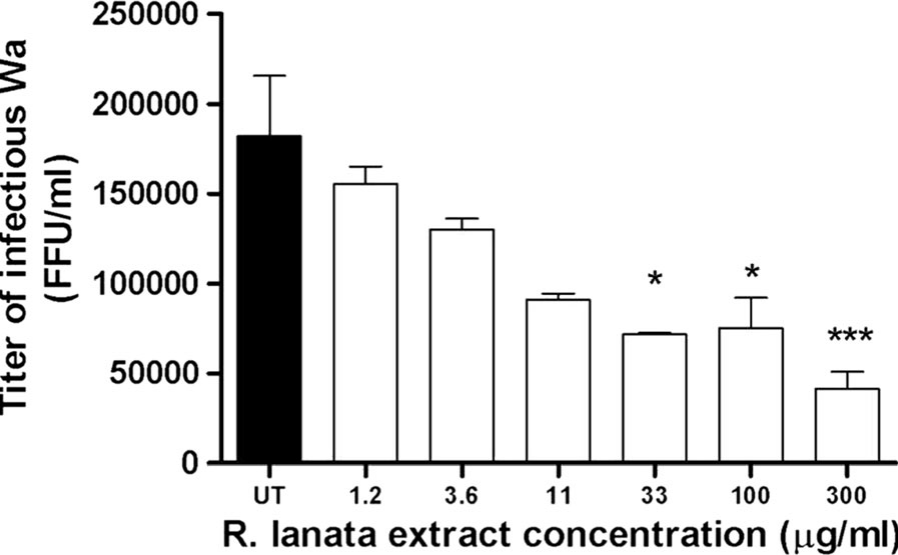
Effect of methanol extract of *R. lanata* on multiple cycles of HRoV-Wa replication. Cells were treated after virus infection at the given concentrations. The titer of HRoV in the treated samples is expressed as focus-forming units per ml (FFU/ml). Error bars represent the SD of the mean of 3 independent experiments. One-way analysis of variance (ANOVA), followed by the Bonferroni test, was used to assess the statistical significance of differences in virus titers. Significance was set at the 95% level. *p < 0.05; ***p < 0.001

To investigate further the extract antiviral efficacy, a virus yield reduction assay was performed by treating cells after viral inoculum, and titrating viral progeny. We chose this experimental setting because represents a stringent kind of test, allowing multiple cycles of replication to occur before measuring antiviral activity. Figure 1 shows that the extract significantly (0.001 < p_ANOVA_ < 0.05) reduced the titer of HRoV progeny in a dose-dependent manner. This result confirms that *R. lanata* methanolic extract affects HRoV replication, likely by targeting a specific step of viral replication.

### *R. lanata* methanolic extract inhibits viral infectivity by targeting the early steps of viral infection

**Fig. 2:**
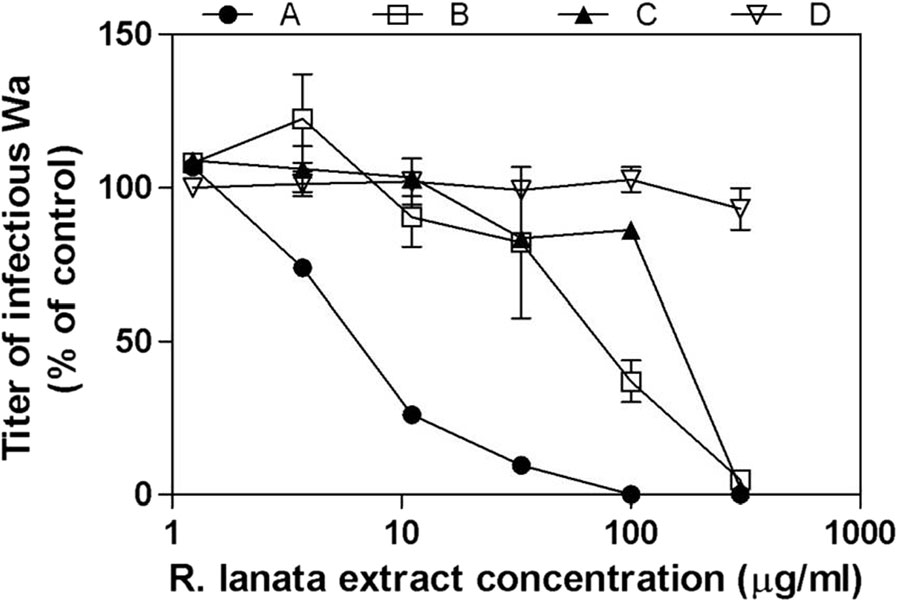
Time-of-addition experiments were performed by treating cells with *R. lanata* methanolic extract for 2 h before infection (*B*), for 1 h during infection (*C*) or immediately after infection (*D*). Control experiments were performed by treating cells both before and during viral inoculum (*A*). The infectivity titers of virus in the treated samples are expressed as a percentage of the titer obtained in theuntreated control. Error bars represent the SD of the mean of 3 independent experiments

**Fig. 3:**
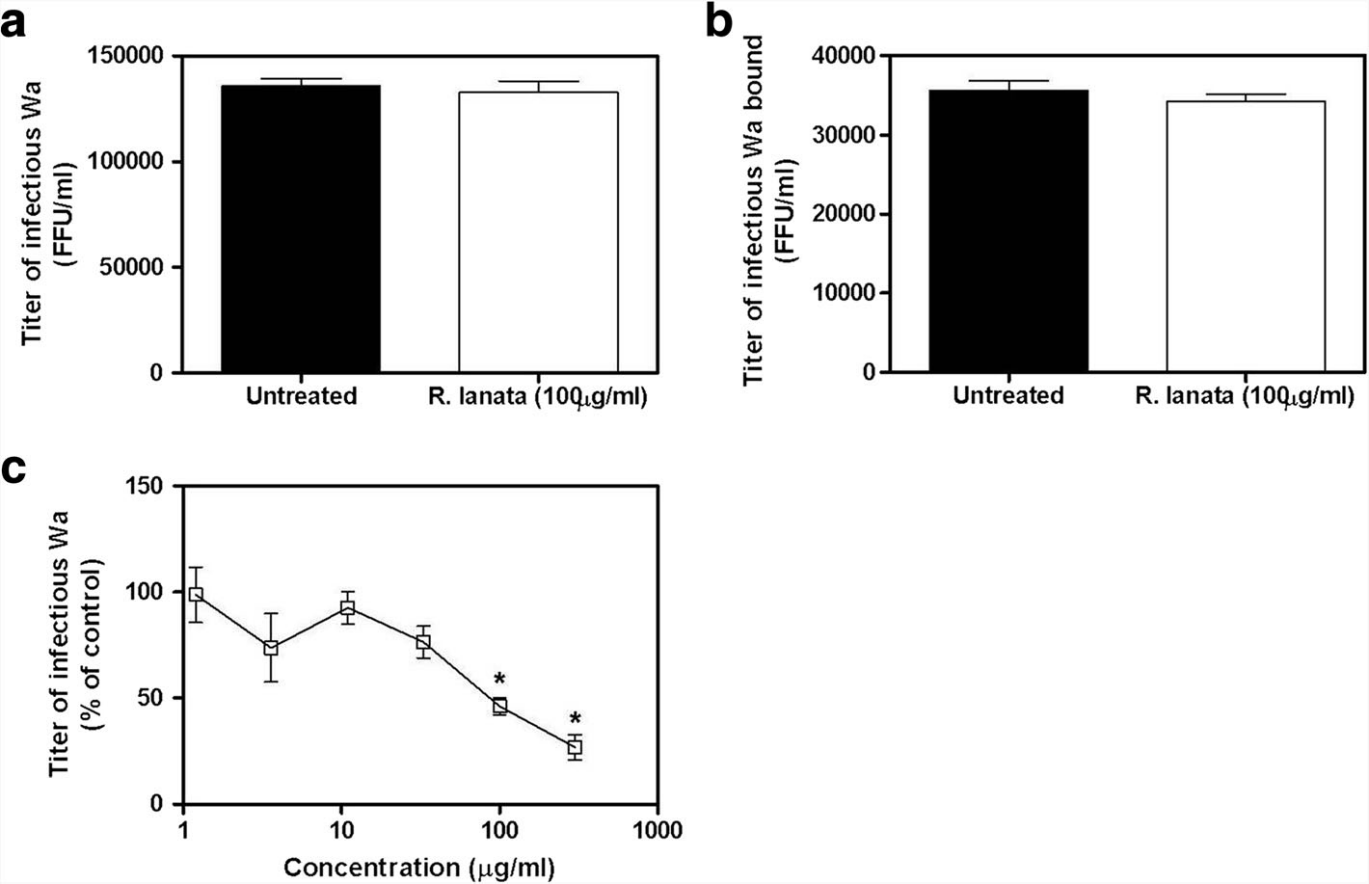
Investigation of the mechanism of anti-HRoV action of *R. lanata* methanol extract. Panel **a** shows the evaluation of the virucidal effect of *R. lanata* methanol extract on infectious HRoV particles. On the *y* axis, the HRoV Wa infectious titers are expressed as focus-forming units per ml (FFU/ml). Panel **b** displays the effect of *R. lanata* methanol extract on Wa binding to the MA104 cell surface; on the *y* axis, the infectious titer of Wa bound to cells is expressed as a % of the titer bound to control MA104 cells in the absence of treatment. Panel **c** shows the effect of *R. lanata* methanol extract on HRoV-Wa entry into MA104 cells; the viral titer measured in the treated samples is expressed as a % of the titer obtained in the untreated control. On the three graphs, error bars represent the SD of the mean of 3 independent experiments.* *p* < 0.05

To investigate the step of viral replication inhibited by the extract, we performed time of addition assays by treating cells with serial dilutions of extract 2 h before infection or during infection or post-infection. The results, depicted in Fig. 2, show that the first two treatment protocols were active while the latter was not, suggesting a probable activity of the extract on the early steps of HRoV infection. To explore this hypothesis, more stringent tests were performed to assess the putative target of the extract (i.e. the viral particle or the virus-cell interactions). Firstly, we assessed the extract ability to directly inactivate the virus particle by incubating HRoV with a high concentration (100 μg/ml) of methanolic extract and then measuring the viral titer at dilutions corresponding at concentrations that are not antiviral when added on cells. Results depicted in Fig. 3a demonstrate that the methanolic extract did not inactivate the virus particles. Having excluded the virus particle as a target of the antiviral activity of the extract, we explored the possibility that the interaction between the viral particles and the cell surface could be affected by the extract. To this aim HRoV-cell binding assays were performed. The results (Fig. 3b) demonstrate that the treatment did not reduce significantly (*p* > 0.05) the titer of virus particles bound to the surface of treated cells, thus suggesting that inhibition occurs at a post-binding stage. To verify this hypothesis, entry assays were performed by treating cells during virus penetration. Figure 3c shows that the *R. lanata* methanolic extract significantly reduced HRoV infectivity in a dose-dependent manner when added immediately after the virus-cell attachment step (i.e. during cell penetration).

## Discussion

Several recent studies demonstrate that plant extracts are deeply explored for their antiviral potential against HRoV [9–14, 27–29].

In this study we show that the methanolic extract of *R. lanata* is endowed with anti-HRoV activity. Although several molecules inside this crude extract can contribute to its effect, each one acting through a specific mechanism, some preliminary conclusions can be drawn on the main mechanism of antiviral action. The treatment with the methanolic extract of *R. lanata* does not alter the ability of HRoV to attach on the host cell surface, as shown by the results of the binding experiments; moreover, the results of the virus inactivation assays rule out a direct antiviral effect of any component of *R. lanata* methanolic extract. By contrast, the results suggest that inhibition takes place at a post-attachment stage, since no antiviral effect is shown when the methanolic extract is added on cells 1 h after the viral inoculum (Fig. 2) - i.e. when the entry process already started and the 30% of the inoculated HRoV particles already entered inside cells [30]. Nevertheless, since the inhibition of entry is not complete it is likely that other, yet unidentified mechanisms, contribute to the overall antiviral activity of the extract. Of note, cells pre-treatment with the extract affects their susceptibility to the HRoV infection (Fig. 2) suggesting that the extract may target cellular functions required for virus replication. Among the major phenolic compounds present in the methanol extract of *R. lanata* hesperidin is known to a have anti-HRoV activity [31] and might be responsible, at least in part, to the anti-HRoV effect of the extract.

## Conclusion

In conclusion, the favorable selectivity index and the anti-HRoV activity against multiple viral strains make the *R.lanata* extract a promising starting material for a bioguided-fractionation aimed at identifying anti-HRoV compounds. Further work remains to be done in order to isolate the active principle, elucidate its mechanism of action and assess its clinical potential.

## Abbreviations

[M-H]: Deprotonated ions of the standard compounds
C.I.: Confidence interval
CC_50_: Half maximal cytotoxic concentration
CPE: Cytopathic effect
DMEM: Dulbecco’s Modified Eagle Medium
EC_50_: Half maximal effective concentration
FBS: Fetal bovine serum
FFU/ml: Focus-forming units per ml
HRhV: Human rhinovirus
HRoV: Human rotavirus
MEM: Eagle’s minimal essential medium
MOI: Multiplicity of infection
MS 2: MRM fragments for the related molecular ions
RSV: Respiratory syncytial virus
RT: Retention time
SI: Selectivity index
